# The Proposed International Dental Congress

**Published:** 1885-09

**Authors:** 


### THE PROPOSED INTERNATIONAL DENTAL CONGRESS.

At the late meeting of the American Dental Association, a proposition was advanced and a committee appointed to report upon a subject that requires careful consideration. The work was done without the discussion and interchange of views that so momentous a matter should demand, and therefore it is the more important that it be taken up in the journals. We refer to the proposition to hold an International Dental Congress during the time of the meeting of the Medical Congress in Washington, in 1887.

Those who read the editorial in the last number of this Journal will comprehend the present status in the medical profession. The work that was done by the committee first appointed has been overturned, and the section of Oral and Dental Surgery has been dropped. A new set of men is in control of the Congress, and, unfortunately, they are not, in all cases, those who are the best representatives of professional standing. Very many of those physicians who were active in the first organization and who are the best known in the medical world have resigned their positions, and have declared that they will have nothing to do with the Congress as it is at present organized. Without their co-operation it is doomed to failure, either positive or comparative.

The object in reconstructing the general committee of the Congress was to place the control and direction of it in the hands of the American Medical Association, and to exclude from membership all societies and physicians who were not members of or loyal to that body. But the Congress has never heretofore been controlled by any one society, and it is foreign to its policy to become the guests of any such body. The quarrel over the organization has become so bitter and the dissension so public, that Sir James Paget, the President of the London Congress of 1881, has written a letter to Dr. I. Minis Hays, of Philadelphia, in which he warns the profession of America that unless these dissensions be soon healed, and the excluded members be accorded a place, the acceptance of the invitation to meet in America will probably be withdrawn, and the Congress called to meet in Berlin. Referring to the method of organization of the Congress of 1878, 1881 and 1884, he says:

"I fully believe that it was understood at Copenhagen that the same course would be pursued in the organization of the Congress at Washington. * * * * Certainly it was not supposed that the Congress would be regulated with any degree of exclusiveness by the members of one medical association, however numerous; and I think it is quite certain that if this had been thought possible, the proposal that the next meeting should be held in the United States would not have been adopted. I am sorry, also, to feel sure that if the Congress be not supported by the eminent men who have now declared that they will take no part in it, the members of the profession in this country who will attend it will be very few. And in this opinion, as well as in all that I have written here, I have the concurrence of several of the most influential of the London Congress with whom, before this writing, I consulted."

Sir William MacCormack, the Secretary-General of the London Congress, writes as follows:

"I am sure it was present to the mind of everyone there (at Copenhagen, 1884), that the invitation was one from the profession of America, and not from any section of it, or any particular medical society in it. Otherwise, I feel pretty certain that Prof. Virchow's invitation to meet on the next occasion in Berlin would have been accepted. Even now it would appear to me wiser to have that invitation renewed, or to meet in some other place, than to have a meeting in America, from which, so far as we may at present judge, many of the men on both sides of the Atlantic would absent themselves."

It may thus be seen that, under the conduct of the men at present in control of the Congress of 1887, it cannot result in anything creditable. There will be very few, if any, delegates in attendance from abroad, and it is fortunate for us that the dental section has been dropped. We desire to repeat very emphatically:—*unless there be a change in the administration of affairs, we as dentists shall only lose in dignity and standing by having anything to do with it.* Should there be the desired re-reconstruction, and things so ordered that such men as Agnew, Bartholow, Gross, Leidy, Yandell, Jacobi, Roosa, Bowditch, Warren, Tiffany, and many others can co-operate, there is little doubt that our section will be readmitted.

We cannot, without serious loss of dignity, sue to the present general committee of the Congress for reinstatement as a section, after being once dropped by them under humiliating circumstances. Nor should we wish such reinstatement, in the face of a probable failure.

Should the section not be re-established, it would, to our mind, be a very questionable act to call an International Dental Congress to meet at the same time, for the following reasons: We probably could not make of it a success. We should be in a very undignified position, as a kind of hanger-on to a Congress from which we had been rudely repulsed. We could have no connection with the Congress, any more than would a meeting of photographers, or commercial drummers, or spindle-shanked bicyclists. We would not even be a part of the tail to the medical kite, for we should have no connection with it. We could not secure foreign attendance, for the dentists of Europe would value their dignity too much to attend such a meeting, even were we so forgetful of our own as to invite them.

The only thing for dentists to do is to pursue the even tenor of their way, doing what they can for the proper reconstruction of the General Committee of the Congress. If this be secured, and the meeting promises to be a success, we shall doubtless be invited to take a part in it. If not, we are lucky to be out of it, for we should present but a sorry spectacle should we labor either for our own re-establishment in the Congress under the present regime, or for the organization of a separate International Dental Congress, and, when we were fully committed, find to our chagrin that an event, which now seems extremely probable, had occurred, and the acceptance of the invitation to meet in America had been withdrawn, and the meeting called for Berlin.

